# The changing burden of malaria and association with vector control interventions in Zambia using district-level surveillance data, 2006–2011

**DOI:** 10.1186/1475-2875-12-437

**Published:** 2013-12-01

**Authors:** Mulakwa Kamuliwo, Emmanuel Chanda, Ubydul Haque, Mercy Mwanza-Ingwe, Chadwick Sikaala, Cecilia Katebe-Sakala, Victor M Mukonka, Douglas E Norris, David L Smith, Gregory E Glass, William J Moss

**Affiliations:** 1 Ministry of Health, National Malaria Control Centre, Lusaka, Zambia; 2 Department of Public Health, Copperbelt University, School of Medicine, Ndola, Zambia; 3 W. Harry Feinstone Department of Molecular Microbiology and Immunology, Bloomberg School of Public Health, Johns Hopkins University, Baltimore, Maryland, USA; 4 Department of Epidemiology, Bloomberg School of Public Health, Johns Hopkins University, Baltimore, Maryland, USA

## Abstract

**Background:**

Malaria control was strengthened in Zambia over the past decade. The two primary interventions for vector control are indoor residual spraying (IRS) and long-lasting insecticide-treated nets (LLINs). Using passive malaria surveillance data collected from 2006 to 2011 through the Zambian District Health Information System, the associations between increased coverage with LLINs and IRS and the burden of malaria in Zambia were evaluated.

**Methods:**

National passive malaria surveillance data from 2006 to 2011 were analysed. A district-level, random-effects model with Poisson regression was used to explore the association between malaria cases and coverage with LLINs and IRS. Malaria cases and LLINs and IRS coverage were mapped to visualize spatiotemporal variation in malaria for each year.

**Results:**

From 2006–2011, 24.6 million LLINs were distributed and 6.4 million houses were sprayed with insecticide. Coverage with LLINs was not uniformly distributed over the study period and IRS was targeted to central and southern districts where malaria transmission was low. LLIN coverage was associated with a reduction in malaria cases, although an increase in the number of malaria cases was reported in some districts over the study period. A high burden of malaria persisted in north-eastern Zambia, whereas a reduction in the number of reported malaria cases was observed in western and southern Zambia.

**Conclusion:**

Enhanced and targeted interventions in north-eastern Zambia where the burden of malaria remains high, as well as efforts to sustain low malaria transmission in the south-west, will be necessary for Zambia to achieve the national goal of being malaria free by 2030.

## Background

An estimated 219 million episodes of malaria and 660,000 malaria deaths occurred worldwide in 2010 [1]. Approximately 80% of malaria episodes and 90% of the deaths were reported from the African continent [1]. International funding for malaria control rose to a peak of USD 1.84 billion in 2012 [1]. These financial commitments to malaria control made increased coverage of four key interventions possible: distribution of long-lasting insecticide-treated nets (LLINs), scale-up of indoor residual spraying (IRS), early diagnosis and treatment with artemisinin-combination therapy, and intermittent preventive treatment in pregnancy (IPTp) [1,2]. Coverage with LLINs and IRS increased rapidly in some countries of sub-Saharan Africa, with household LLIN ownership across the region reaching 53% by 2012 and IRS protecting 11% of the population at risk [1].

Zambia has a history of highly endemic malaria transmission with varying levels of malaria control efforts over the past 50 years [3]. In the past decade, malaria control was strengthened, leading to substantial increases in coverage following the availability of external funding [4]. Zambia was successfully awarded the Global Fund Rounds 1, 4 and 7 for malaria control and was successful in obtaining financial support from the US President’s Malaria Initiative, the UK’s Department for International Development and the World Bank to scale-up LLIN and IRS coverage. However, through 2011, malaria remained endemic in all of the country’s 72 administrative districts [3,5].

The two primary interventions for vector control in Zambia are IRS and LLINs. Using passive malaria surveillance data collected from 2006 to 2011 through the Zambian District Health Information System (DHIS), the district-level associations between IRS and LLIN coverage and the number of reported malaria cases were evaluated.

## Methods

### Study area

Zambia is a land-locked country in southern Africa, bordering eight malaria-endemic countries: Angola, Botswana, Democratic Republic of Congo, Malawi, Mozambique, Namibia, Tanzania, and Zimbabwe. The population of Zambia is approximately 13 million, with two-thirds of the population residing in rural areas [5]. The country has a land area of 752,618 sq km, with an overall population density of 17 persons per sq km. The average life expectancy in Zambia is 49 years for men and 50 years for women [6].

Zambia is stratified into three malaria epidemiological zones: a low-transmission region in south-eastern Zambia with parasite prevalence <1%; a low stable-transmission zone in north-western/south-central Zambia with a parasite prevalence of 10%; and a high-transmission zone in northern and eastern Zambia with a parasite prevalence of >20% [3]. Zambia experiences three distinct seasons, with a rainy season extending from November to May during which malaria transmission peaks, a cool dry season from late May to August, and a hot dry season from September to November. Persistent year-round malaria transmission occurs in focal locations across the country [3].

Malaria in Zambia is almost entirely caused by *Plasmodium falciparum* infection (~98%), with a low frequency of infections due to *Plasmodium malariae* and *Plasmodium ovale*, and little or no transmission of *Plasmodium vivax*[3]. The main malaria vectors in Zambia are *Anopheles gambiae s.s., Anopheles arabiensis* and *Anopheles funestus s.s*[7,8]. The epidemiology of malaria in Zambia is the result of two major forces: geographic, climatic and social features that establish ecological conditions conducive or restrictive to malaria transmission, and the introduction and scaling-up of malaria control interventions. Both forces alter malaria transmission and infection, as well as associated morbidity and mortality.

### Malaria morbidity and mortality by district

Annual reported, district-level, aggregated malaria case data were obtained from the Zambian DHIS, the equivalent of the national health management information system. DHIS data are collected at heath facilities (including district hospitals and health centres) in paper form and are sent to the District Health Office (DHO) for data capture and validation in the DHIS. The DHO transmits the district-level data to the provincial office for further processing and aggregation for the province. The consolidated provincial data are then transmitted to the National Malaria Control Centre (NMCC), which in turn provides feedback and technical support to the provinces, districts and health facilities.

Severe malaria, a set of clinical and laboratory parameters associated with an increased risk of death in the presence of *P. falciparum* parasitaemia, was reported from hospital admission records [9,10]. Malaria episodes, including deaths, were reported by age group: <five and ≥ five years. From 2006 to 2008, only the number of malaria episodes was reported. Clinical and confirmed malaria by rapid diagnostic test (RDT) or microscopy were reported separately from 2009 to 2011.

### Spatial and population data

District polygon boundaries (shape files) were obtained from the Zambian Ministry of Environment and Statistics in Lusaka. For 2010, demographic and housing data were obtained from the Zambian Bureau of Statistics (ZBS) [11]. District-level demographic data were projected for 2006, 2007, 2008, 2009, and 2011 using an exponential population growth model based on ZBS reports for 2000 and 2010. The number of houses in each district was projected based on 2000 and 2010 estimates, assuming linear growth. These estimates served as denominators for the calculation of rates [11,12]. Morbidity and mortality rates attributable to malaria were calculated by age group (<five and ≥ five years) per 1,000 population based on data from the DHIS and population estimates. This study was deemed not be human subjects research by the Johns Hopkins Bloomberg School of Public Health Institutional Review Board.

### IRS and LLIN coverage by district

Data on IRS coverage were captured using daily spray forms that were annually consolidated at the NMCC. Data were also available on the number of LLINs distributed annually through various distribution channels, including antenatal and under-five clinics and mass vaccination campaigns. Both IRS and LLIN coverage data were aggregated at the district level. LLIN rates were calculated as the number of nets per two household residents and an assumed three-year life span of LLINs [13-15]. IRS rates were calculated per 1,000 houses. IRS and LLIN coverage rates also were estimated at the provincial level.

### Statistical analysis

Poisson regression was used to explore the association between the burden of malaria, as measured by the total number of malaria cases either diagnosed clinically or confirmed by microscopy or RDT, and LLIN and IRS coverage at the district level using 2006 as the baseline year. The random effect of the district variable was used to model variation between districts and by year. All statistical analyses were performed using STATA 11 (STATA Corp. 2003, College Station, TX, USA).

### Hotspot analysis

Annual malaria cases and annual IRS and LLIN coverage data were linked with district shape files. Spatial autocorrelation was tested for total yearly malaria cases using incremental spatial autocorrelation. The local Getis-OrdGi* statistic [16] was used to generate hotspot maps for malaria prevalence and IRS and LLIN coverage from 2006 to 2011. The local Getis-OrdGi* statistic compares the local mean rate (the rates for a district and its nearest neighbouring districts) to the global mean rate (the rates for all districts), producing a z-score and p-value for each district, reflecting whether the differences between the local and global means are statistically significant [17]. A statistically significant positive z-score indicates a hotspot for high rates. Similarly, a statistically significant negative z-score for a district indicates local spatial clustering of low rates [16,18,19]. To identify persistent malaria hotspots, the number of malaria cases in each year from 2007 to 2011 was subtracted from the number of cases in 2006. This change in the number of malaria cases for each year also was used in a hot spot analysis. These analyses were conducted using ArcGIS 10 (ESRI, Redlands, CA, USA).

## Results

### Changes in the burden of malaria

**Figure 1:**
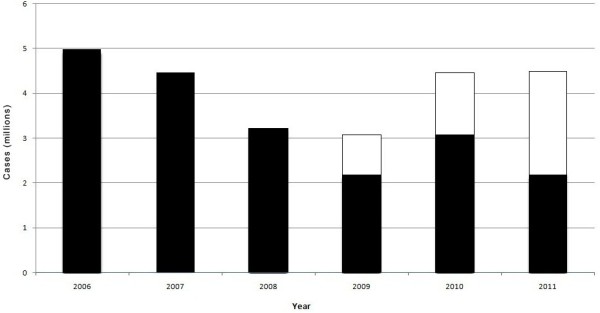
**Trends in malaria cases and method of diagnosis in Zambia, 2006 to 2011.** (Black bars indicate clinical cases, open bars indicate cases confirmed by microscopy or RDT).

In 2006, 4.98 million cases of malaria were reported in Zambia. Using 2006 as baseline, a sharp decline in the number of reported cases was observed through 2009 followed by an increase in malaria cases in 2010 that continued through 2011 (Figure 1). In 2011, a total of 4.54 million cases of malaria were reported (Figure 1). From 2008 to 2011, an increasing number of districts reported low malaria prevalence, ranging from < ten to 100 cases per 1,000 population. Despite this improvement, 37 districts reported resurgence (a return towards the 2006 baseline level or higher) of malaria in 2011 [20].

**Figure 2:**
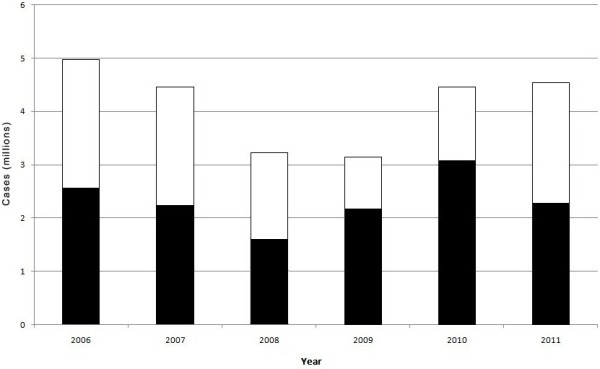
**Malaria in Zambia by age groups, 2006 to 2011.** (Black bars indicate cases < five years, open bars indicate cases ≥ five years).

No clear trend in the number of malaria cases among children < five years and individuals ≥ five years was apparent (Figure 2). From 2006–2011, the rate of severe malaria was more than five times higher among children < five years (50 severe malaria cases per 1,000 population) compared with those ≥ five years old (nine severe malaria cases per 1,000 population) (Table 1). Mean mortality rates were more than five times higher among children < five years (1.3 deaths per 1,000 population) compared with those ≥ five years (0.2 deaths per 1,000 population) (Table 1).

**Table 1:** Reported national annual cases for malaria indicators, 2006 to 2011

**Year**	**2006**	**2007**	**2008**	**2009**	**2010**	**2011**	**Mean**
Malaria prevalence/1,000 persons	448	398	269	260	352	392	353
Malaria prevalence <5 years/1,000 persons	1,220	1,033	684	703	770	817	871
Malaria prevalence ≥5 years/1,000 persons	265	246	170	157	256	263	226
Severe malaria/1,000 persons	23	19	13	14	19	16	17
Severe malaria <5 years/1,000 persons	73	61	38	43	46	40	50
Severe malaria ≥5 years/1,000 persons	11	9	7	7	10	9	8.98
Malaria related mortality/1,000 persons	0.53	0.47	0.30	0.34	0.37	0.35	0.39
Malaria related mortality <5 years/1,000 persons	1.55	1.43	0.81	1.13	1.80	0.96	1.28
Malaria related mortality ≥5 years/1000 persons	0.29	0.24	0.17	0.16	0.32	0.17	0.23
Clinical malaria cases (%)	-	-	-	69	69	50	63
Confirmed* malaria cases (%)	-	-	-	31	31	50	37
LLIN distribution/per two household residents	129	347	413	456	344	600	382
IRS coverage/1,000 houses	137	156	232	277	465	628	316

*Confirmed by microscopy or RDT.

Reporting of both clinical diagnoses and confirmed cases by RDT or microscopy started in Zambia from 2009. Only half (50.2%) of the reported cases were confirmed by either microscopy or RDT in 2011 (Figure 1).

### LLIN and IRS coverage

**Figure 3:**
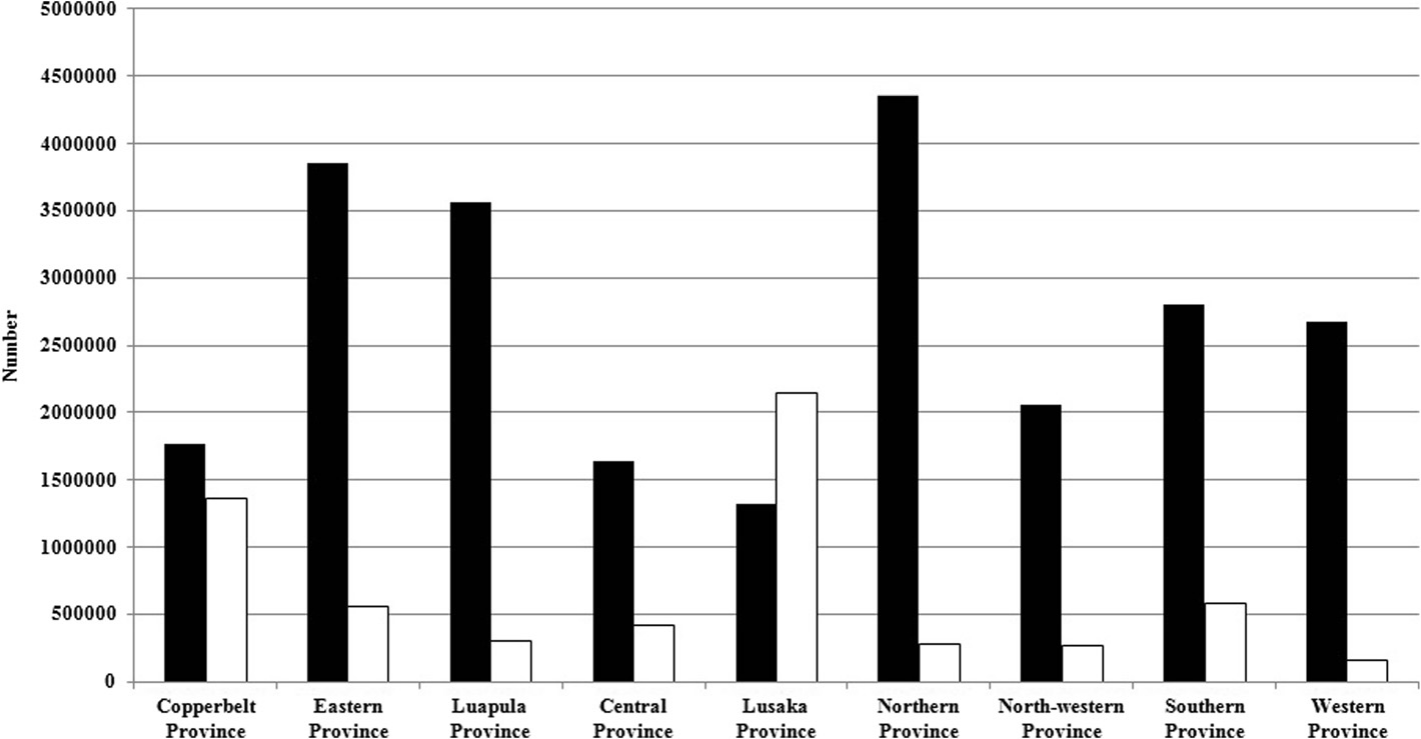
**Distribution of IRS and LLIN by province, 2006 to 2011.** (Black bars indicate LLINs, open bars indicate IRS).

Between 2006 and 2011, 24.06 million LLINs were distributed and 6.07 million houses were covered by IRS throughout the country (Table 1). From 2006 to 2011, LLIN and IRS coverage increased 4.7-fold and 4.6-fold, respectively (Table 1). IRS began in 15 districts in 2006 and 2007 and was expanded to 28 districts in 2008 and 2009, 54 districts in 2010 and 69 districts in 2011 in Zambia. Mean IRS coverage between 2006 and 2011 was 316 per 1,000 houses (Table 1); however, wide variation in IRS and LLIN coverage existed at the district level (Figure 3).

### Associations between vector control activities and the burden of malaria

The decline in the burden of malaria was associated with LLIN coverage but not IRS coverage at the district level. The prevalence of malaria was 9% lower in those districts having ≥ one LLIN for two persons compared to those districts with less than one LLIN for two persons (Table 2). The impact of LLIN was greater for young children as districts with ≥ one LLIN for two persons were associated with a 16% reduction in the prevalence of malaria for children < five years compared with a 7% reduction in individuals ≥ five years (Table 3). The prevalence of severe malaria was 11% lower in those districts having ≥ one LLIN compared to those districts with less than one LLIN for every two persons (Table 2). The impact of LLIN on severe malaria was also greater for young children as districts with ≥ one LLIN for two persons were associated with a 15% reduction in severe malaria in children < five years compared with an 8% reduction in individuals ≥ five years (Table 3). No overall associations were observed between mortality attributed to malaria and coverage with LLIN (Table 2), although a 10% reduction in mortality among individuals ≥ five years was associated with ≥ one LLIN for two persons (Table 3).

**Table 2:** Impact of IRS and LLIN on the prevalence of malaria in Zambia, 2006 to 2011

	**Total prevalence**	**Severe malaria**	**Deaths due to malaria**
	PRR* (95% CI)	**PRR (95% CI)**	**PRR (95% CI)**
**IRS** (per 1,000 household coverage)			
0	1	1	1
<500	1.19 (1.19–1.20)	1.33 (1.32–1.34)	1.12 (1.07–1.17)
≥500	1.49 (1.49–1.50)	1.49 (1.48–1.50)	1.54 (1.46–1.62)
**LLIN** (per two household residents coverage)			
<1	1	1	1
≥1	0.91 (0.90–0.91)	0.89 (0.88–0.89)	0.96 (0.92–1.00)
**Year**			
2006	1	1	1
2007	0.89 (0.89–0.90)	0.87 (0.86–0.87)	0.94 (0.90–0.97)
2008	0.61 (0.61–0.62)	0.58 (0.57–0.58)	0 .54 (0.51–0.57)
2009	0.58 (0.57–0.58)	0.63 (0.62–0.63)	0.61 (0.59–0.65)
2010	0.72 (0.71–0.72)	0.74 (0.73–0.74)	0.61 (0.58–0.65)
2011	0.74 (0.73–0.74)	0.60 (0.59–0.60)	0.55 (0.52–0.58)

*PRR = Prevalence rate ratio.

**Table 3:** Impact of IRS and LLIN on the prevalence of malaria by age group in Zambia, 2006 to 2011

	**Malaria prevalence**		**Severe malaria**		**Death**	
	**<5 years**	**≥5 years**	**<5 years**	**≥5 years**	**<5 years**	**≥5 years**
	PRR* (95% CI)	**PRR (95% CI)**	**PRR (95% CI)**	**PRR (95% CI)**	**PRR (95% CI)**	**PRR (95% CI)**
**IRS** (per 1,000 household coverage)						
0	1	1	1	1	1	1
<500	1.13 (1.13–1.14)	1.27(1.26–1.27)	1.28 (1.26–1.29)	1.33 (1.32–1.35)	1.28 (1.21–1.36)	0.96 (0.90–1.03)
≥500	1.36 (1.35–1.36)	1.57(1.57–1.58)	1.38(1.36–1.39)	1.58 (1.56–1.60)	1.72 (1.60–1.84)	1.24 (1.14–1.34)
**LLIN** (per two household residents coverage)						
<1	1	1	1	1	1	1
≥1	0.84 (0.84–0.85)	0.93 (0.93–0.94)	0.85 (0.84–0.85)	0.92 (0.91–0.93)	1.03 (0.97–1.10)	0.90 (0.84–0.96)
**Year**						
2006	1	1	1	1	1	1
2007	0.88 (0.88–0.89)	0.91 (0.90–0.91)	0.87 (0.87–0.88)	0.86 (0.86–0.87)	1.09 (1.04–1.15)	0.76 (0.72–0.80)
2008	0.61 (0.61–0.62)	0.62 (0.62–0.63)	0.55 (0.55–0.56)	0.63 (0.62–0.64)	0.49 (0.46–0.52)	0.59 (0.55–0.63)
2009	0.65 (0.65–0.66)	0.55 (0.55–0.56)	0.68 (0.67–0.69)	0.62 (0.62–0.63)	0.66 (0.61–0.70)	0.59 (0.55–0.63)
2010	0.69 (0.68–0.69)	0.79 (0.78–0.79)	0.67 (0.66–0.68)	0.76 (0.75–0.77)	0.56 (0.52–0.61)	0.69 (0.65–0.75)
2011	0.67 (0.67–0.68)	0.75 (0.75–0.76)	0.53 (0.53–0.54)	0.63 (0.62–0.64)	0.47 (0.43–0.50)	0.55 (0.51–0.60)

*PRR = Prevalence rate ratio.

### Hotspots

**Figure 4:**
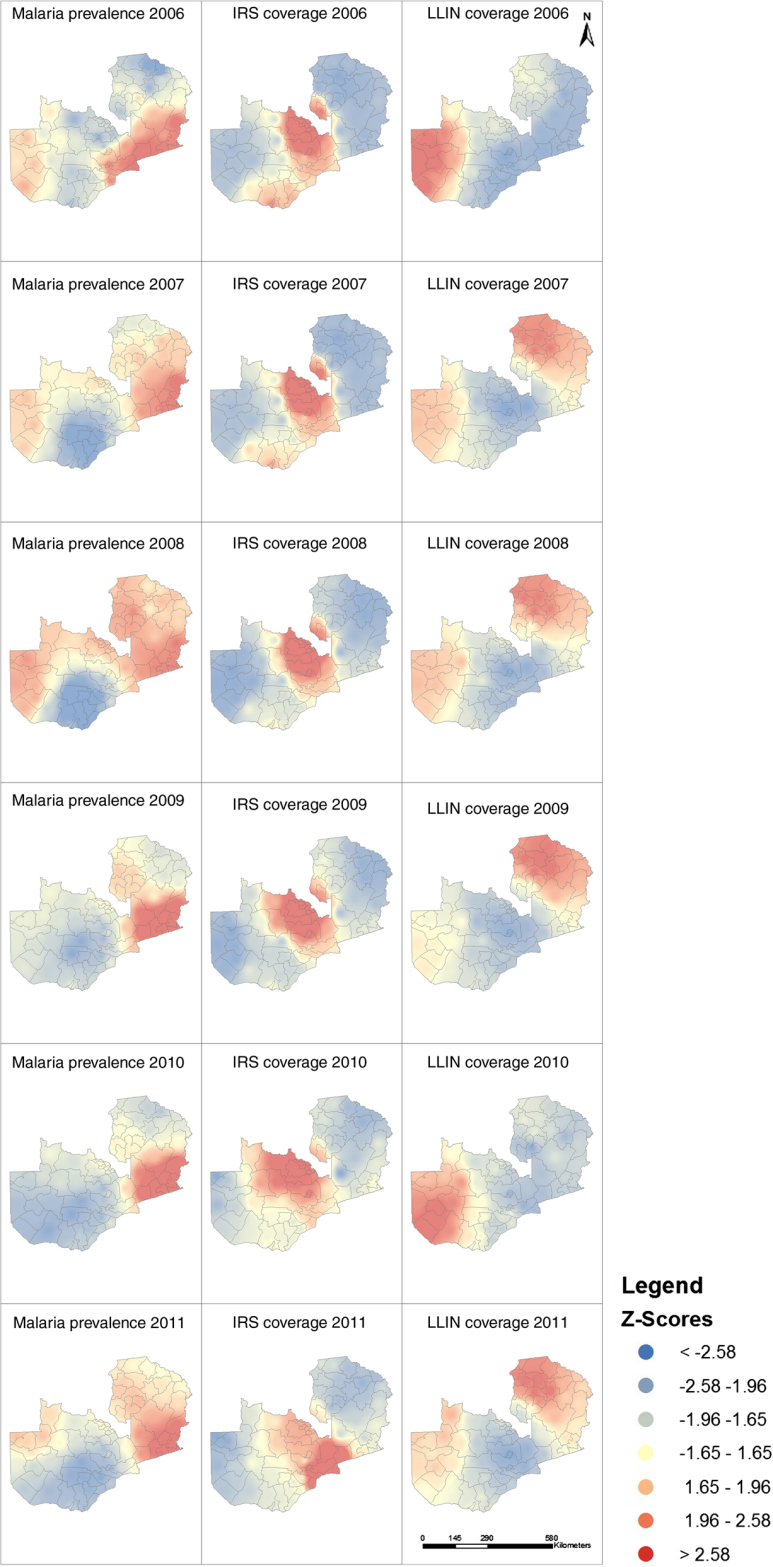
Maps of malaria prevalence, LLIN distribution and IRS distribution.

**Figure 5:**
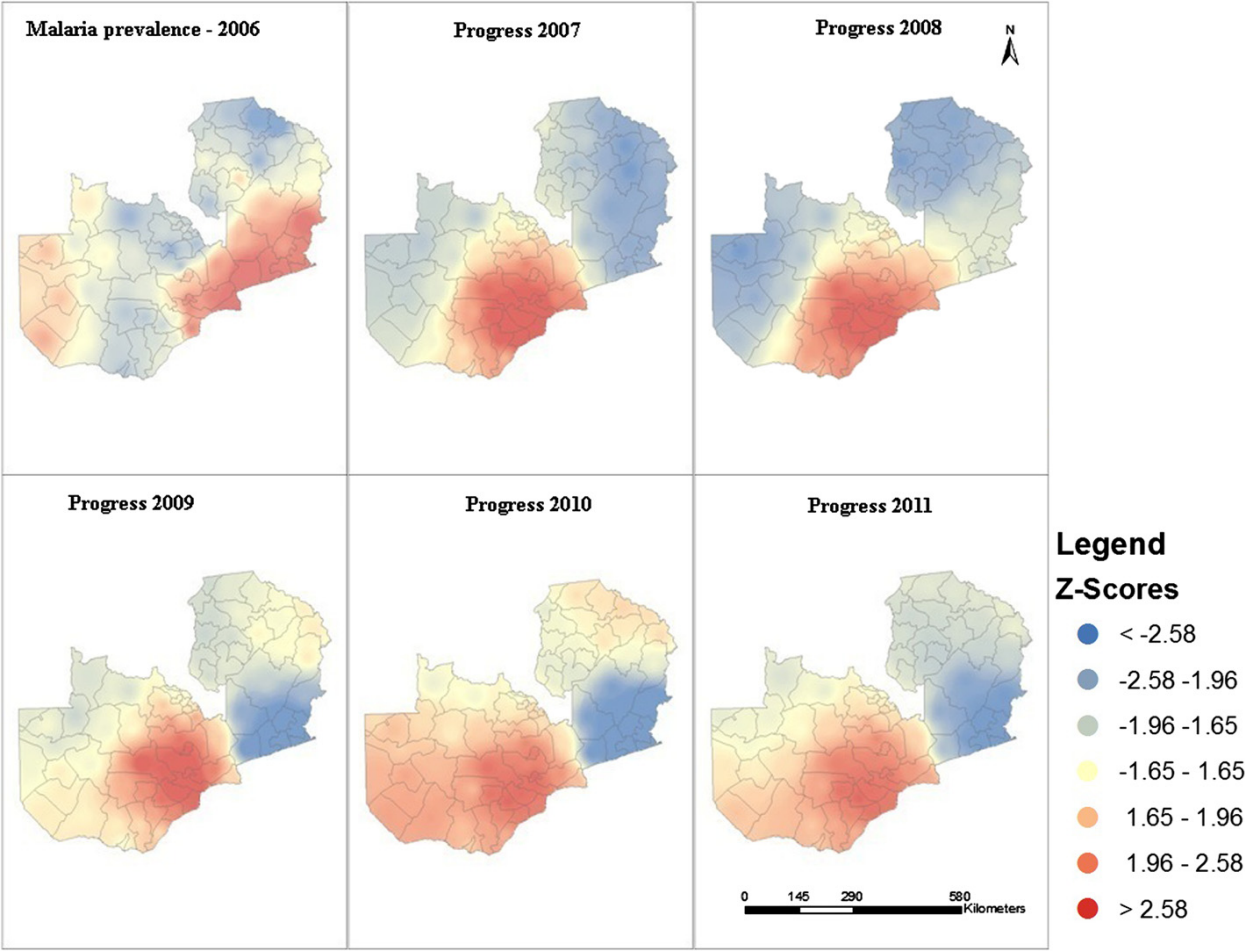
Changes in the prevalence of malaria in Zambia based on 2006 levels.

Applying hotspot analysis to malaria prevalence revealed statistically significant (p <0.01; z >2.58) hotspots that shifted during the study period (Figure 4). From 2006 to 2008, malaria hotspots were identified in both western and eastern districts of Zambia. Decreased numbers of cases were reported in western districts in 2009 and 2010, and a few districts in north-western Zambia reported hotspots in 2011 (Figure 4). South-eastern districts in Zambia, sharing a border with Mozambique and Malawi, were continuously identified as hotspots (Figure 4). In 2006, LLIN coverage was first implemented in western Zambia. Distribution decreased in western Zambia in 2007 and higher coverage was reported in the eastern districts until 2008. Higher LLIN coverage was reported in the north-eastern districts in 2009, shifting to western Zambia in 2010 and north-eastern Zambia in 2011 (Figure 4). Districts in the central Zambia never achieved the highest LLIN coverage (Figure 4) but IRS coverage was highest in the central districts from 2006 to 2011 (Figure 4). Maps of changes in malaria prevalence revealed that districts in north-eastern Zambia persisted as malaria hotspots despite national declines in malaria prevalence following control interventions (Figure 5). During this same time period, the burden of malaria was markedly reduced in southern districts.

## Discussion

Zambia made much progress in reducing the burden of malaria between 2006 and 2011, in part through widespread coverage with vector control interventions. This reduction in the burden of malaria was associated with LLIN coverage at the district level. However, malaria remains a major cause of morbidity and mortality in parts of the country, and particularly among children younger than five years of age. After six years of malaria control interventions, both resurgent and persistently stable malaria hotspots were observed in the north-eastern districts of Zambia. Additional malaria control strategies will be needed to reduce the burden of malaria in these regions.

Overall, Zambia achieved distribution of one LLIN for every two persons. However, LLINs may lose effectiveness with the emergence of insecticide resistance [5,8]. In 2010, significant resistance to deltamethrin, permethrin and DDT was detected in both *An. gambiae s.s*. and *An. funestus s.s.* in several districts in Zambia [7]. Vector and insecticide resistance mapping in malaria hotspots could help guide vector control strategies. Larval control has never been widely implemented in Zambia. However, with the emergence of insecticide resistance, larval control through environmental management and larviciding could provide alternatives for vector control in some areas. The selection of larvicides, insecticides for IRS and deployment of LLINs must be guided by detailed knowledge of vector species, their biting and oviposition behaviours and patterns of resistance.

High coverage with IRS targeted a subset of districts in central Zambia, with little extension into the southwest of the country, and was conducted in mostly urban areas. Further progress will require additional strategies, with a focus on north-eastern districts that consistently reported significant and stable malaria hotspots [21].

All countries sharing a border with Zambia have endemic malaria. Cross-border collaborations with neighbouring countries should be strengthened, such as the recent Zambia Zimbabwe Cross-Border Malaria Initiative [3,22]. Persistently high malaria transmission along the Mozambique and Malawi borders indicates the need for additional cross-border collaborations, with monitoring of population movement and malaria transmission. The low level of malaria transmission and decline in the number of malaria cases in southern Zambia, which shares a border with Namibia, Botswana and Zimbabwe, demonstrates the unprecedented achievement of the National Malaria Control Programme.

This study has several limitations, most notably the fact that these analyses were based on routinely collected data within the DHIS with the potential for both over and under reporting of malaria cases. Secular changes in reporting could have biased prevalence estimates and errors could have been introduced as data were aggregated at higher levels of the health information system. However, data accuracy and completeness were not systematically assessed. Many cases reported in the DHIS were not confirmed by RDT and microscopy but were based on clinical signs and symptoms, with the potential for misclassification. Both RDT and microscopy have limited sensitivity and specificity, and are particularly likely to misclassify individuals with low levels of parasitaemia. The observed associations between LLIN and IRS coverage and the burden of malaria were ecologic and not at the level of individuals.

Sustained efforts are critical to maintain the gains that have already been achieved and further progress may bring malaria transmission to near-zero in all four districts that reported fewer than ten cases per 1,000 population in 2011, thus creating malaria-free zones in Zambia. Enhanced and targeted interventions in north-eastern Zambia where the burden of malaria remains high, as well as sustaining low malaria transmission in the southwest, will be necessary for Zambia to achieve the national goal of being malaria free by 2030.

## Competing interests

The authors declared that they have no competing interests.

## Authors’ contributions

MK conceived the idea and contributed to the drafting of the manuscript. UH conceived the idea, conducted data analysis and drafted the manuscript. MMI provided the DHIS data. CHS and CKS coordinated the implementation of IRS and LLINs, respectively. VMM, EC, DLS, DEN and GEG critically reviewed the manuscript and contributed to the drafting of the manuscript. UH and WJM interpreted the results and drafted the final version of the manuscript. All authors read and approved the final manuscript.
